# Outcomes of critically ill patients with acute kidney injury in COVID-19 infection: an observational study

**DOI:** 10.1080/0886022X.2021.1933530

**Published:** 2021-05-30

**Authors:** Rodrigo Bezerra, Flávio Teles, Polyana Bezerra Mendonca, Tedla Damte, Andrew Likaka, Edyniesky Ferrer-Miranda, Jones Oliveira de Albuquerque, José Luiz de Lima Filho

**Affiliations:** a Keizo Asami Laboratory of Immunopathology, Federal University of Pernambuco, Recife, Brazil; b PROCAPE, University of Pernambuco, Recife, Brazil; c Clinical Medicine Department, Federal University of Alagoas, Maceio, Brazil; d Federal University of Pernambuco, Recife, Brazil; e Health department, UNICEF, Lilongwe, Malawi; f Federal Rural University of Pernambuco, Recife, Brazil; g Department of Statistics and Informatics, Federal Rural University of Pernambuco, Recife, Brazil; h Department of Biochemistry, Federal University of Pernambuco, Recife, Brazil

**Keywords:** COVID-19, acute kidney injury, mortality risk, dialysis, renal failure, critical care

## Abstract

**Background:**

Early reports indicate that AKI is common during COVID-19 infection. Different mortality rates of AKI due to SARS-CoV-2 have been reported, based on the degree of organic dysfunction and varying from public to private hospitals. However, there is a lack of data about AKI among critically ill patients with COVID-19.

**Methods:**

We conducted a multicenter cohort study of 424 critically ill adults with severe acute respiratory syndrome (SARS) and AKI, both associated with SARS-CoV-2, admitted to six public ICUs in Brazil. We used multivariable logistic regression to identify risk factors for AKI severity and in-hospital mortality.

**Results:**

The average age was 66.42 ± 13.79 years, 90.3% were on mechanical ventilation (MV), 76.6% were at KDIGO stage 3, and 79% underwent hemodialysis. The overall mortality was 90.1%. We found a higher frequency of dialysis (82.7% versus 45.2%), MV (95% versus 47.6%), vasopressors (81.2% versus 35.7%) (*p* < 0.001) and severe AKI (79.3% versus 52.4%; *p* = 0.002) in nonsurvivors. MV, vasopressors, dialysis, sepsis-associated AKI, and death (*p* < 0.001) were more frequent in KDIGO 3. Logistic regression for death demonstrated an association with MV (OR = 8.44; CI 3.43–20.74) and vasopressors (OR = 2.93; CI 1.28–6.71; *p* < 0.001). Severe AKI and dialysis need were not independent risk factors for death. MV (OR = 2.60; CI 1.23–5.45) and vasopressors (OR = 1.95; CI 1.12–3.99) were also independent risk factors for KDIGO 3 (*p* < 0.001).

**Conclusion:**

Critically ill patients with SARS and AKI due to COVID-19 had high mortality in this cohort. Mortality was largely determined by the need for mechanical ventilation and vasopressors rather than AKI severity.

## Introduction

The coronavirus pandemic, still ongoing, has brought a great challenge to the medical profession, especially for intensivists and nephrologists, who must treat serious complications of the disease, such as severe acute respiratory syndrome (SARS) and acute kidney injury (AKI) [1,2]. Nephrologists have witnessed that, as in other infectious diseases, acute kidney injury is a frequent complication of SARS-CoV-2, especially in its most severe forms. Current data demonstrate a prevalence of up to 56.9% of acute kidney injury among hospitalized patients with SARS-CoV-2 and mortality that varies widely (23.8–97.2%) depending on the characteristics of the sample [3,4]. It has been suggested that COVID-19 associated AKI has a worse prognosis in comparison to other AKI etiologies [5]. However, there are still few epidemiological data on the outcomes of critically ill patients with AKI during COVID-19 infection. This study was conducted to describe the clinical characteristics and risk factors for death among critically ill patients who developed AKI during infection with the coronavirus and who were treated in six public ICUs in a Brazilian city.

## Material and methods

### Study design, setting, and participants

We carried out a multicenter retrospective cohort study that included patients older than 18 years of age diagnosed with the severe acute respiratory syndrome (SARS) and AKI, both due to SARS-CoV-2, admitted to intensive care units (ICUs). A local ethics committee reviewed and approved this research (CAAE: 33452820.8.0000.5192). The STROBE (Strengthening the Reporting of Observational Studies in Epidemiology) guideline recommendations were used as a reference [6]. We excluded patients with chronic kidney disease, which was defined as a report or laboratory evidence of previous renal dysfunction (creatinine clearance estimated by CKD-EPI at <60 mL/min/1.73 m^2^, need for dialysis prior to hospitalization or kidney transplantation), and patients who were transferred from the hospital during follow-up. All assistance was provided by a group of nephrologists who belonged to the same team and followed the same AKI protocols.

Data were extracted from the medical records of six tertiary and referral hospitals of the Local Government for the treatment of COVID-19 in the city of Recife, Brazil, from 8 March to 18 August 2020. Data were collected by two nurses with experience in nephrology and intensive care and one nephrologist. We used telephone follow-up calls to contact patients after hospital discharge until 28 November 2020.

### Exposures

#### Demographic data and comorbidities

Demographic data and other variables were included, such as time of hospitalization, comorbidities, AKI severity, RRT requirement, time on dialysis, dialysis modality (short or prolonged hemodialysis), use the need of mechanical ventilation, use need of vasopressors, highest plasma potassium level, and value of positive end-expiratory pressure (PEEP) at the time of nephrological assessment. The quick Sepsis Related Organ Failure Assessment (qSOFA) was calculated using data from the medical record at admission to the ICU. Comorbidities assessed (systemic arterial hypertension, diabetes mellitus, obesity, chronic obstructive pulmonary disease, and heart failure) were collected from patient medical records.

### Diagnosis of COVID-19

The diagnostic criteria for COVID-19 were fever (temperature ≥37.3 °C) or respiratory symptoms, leukopenia (<4.0 × 10³/µL) or lymphopenia (<1.0 × 10³/µL), and chest tomography with characteristic pulmonary findings. Patients with two of the above criteria underwent RT-PCR on a specimen collected via nasopharyngeal swab for SARS-CoV-2. SARS was defined as a flu-like syndrome with dyspnea, respiratory distress, or persistent chest pressure or oxygen saturation below 95% in room air. Only patients with SARS and confirmation of SARS-CoV-2 infection by RT-PCR were included in the sample.

### Definitions of AKI and recovery

AKI was defined using the Kidney Disease Improving Global Outcomes (KDIGO). AKI severity was staged according to the same criteria [7]. The KDIGO classification was performed at the time of the first assessment by the nephrologist. We used the lowest inpatient serum creatinine as a reference for AKI [8]. Urine output was included in KDIGO classification. The etiology of AKI was defined according to its temporal relationship with well-established aggressive agents and divided into hypovolemic (clinical observation of a rapid decrease in serum creatinine with convergence to the baseline level within 72 h after fluid replacement associated with a clinical presentation suggestive of dehydration), nephrotoxic (temporal relationship with the use of nephrotoxic drugs or radiological contrast) secondary to sepsis (characterized by the simultaneous presence of Sepsis-3 definition and AKI) and obstructive (related to urinary tract obstruction) [9]. Complete renal recovery was achieved when serum creatinine reached a value below the level determined in AKI stage 1, and partial renal recovery was defined as a fall in AKI stage [10]. The progression to chronic kidney disease (CKD) from AKI occurred when creatinine did not reach the criteria of recovery and kidney failure with replacement therapy (KFRT) when the patient remained on dialysis for more than three months [11].

### Dialysis related variables

Dialysis indications were azotemia with uremic symptoms (usually with urea >150 mg/dL), oliguria refractory to diuretics, hyperkalemia refractory to drug treatment (K^+^ > 6.0 mEq/L), hypervolemia and metabolic acidosis (pH < 7.20 and serum bicarbonate < 16 mEq/L in arterial blood). Dialysis was interrupted in situations of severe hemodynamic instability after the beginning of the procedure. Prolonged hemodialysis was defined as hemodialysis sessions with a blood flow rate of 200 mL/min and a dialysate flow rate of 300 mL/min, with duration ranging from 5 to 12 h, and was indicated in all patients who developed hypotension refractory to volume expansion requiring the use of vasoactive drugs. Short hemodialysis was defined as 2- to 4-h sessions with blood flow rates between 300–400 mL/min and dialysate flow rates of 500–800 mL/min. High-flow dialyzers with a surface area of 1.8 m^2^ were used. The sodium concentration, temperature, and ultrafiltration volume were determined according to the nephrologist’s criteria. In patients who did not have contraindications for anticoagulation, unfractionated bolus heparin was used at a dose of 75 U/kg body weight every 3 h.

### Outcomes

The primary outcome was in-hospital mortality. The secondary outcomes were AKI stage, kidney failure with replacement therapy (KFRT), dialysis method, renal recovery, and mechanical ventilation (MV).

### Analytic method

Numerical variables are presented as mean ± standard deviation or median with the interquartile range depending on the result of the Kolmogorov–Smirnov normality test. To test associations between variables and death, we used Student’s *t*-test for continuous variables and the Chi-square test for categorical variables. Logistic regression was performed to analyze associations with death and severe AKI (KDIGO stage 3). Only the more significant variables in univariate analysis were included in logistic regression. Some variables were excluded from regression because of collinearity (sepsis and PEEP). The significance level adopted was *p* < 0.05, and the confidence interval was 95%. All statistical analyses were performed using SPSS software (version 22).

## Results

### Patient characteristics

**Figure 1.**
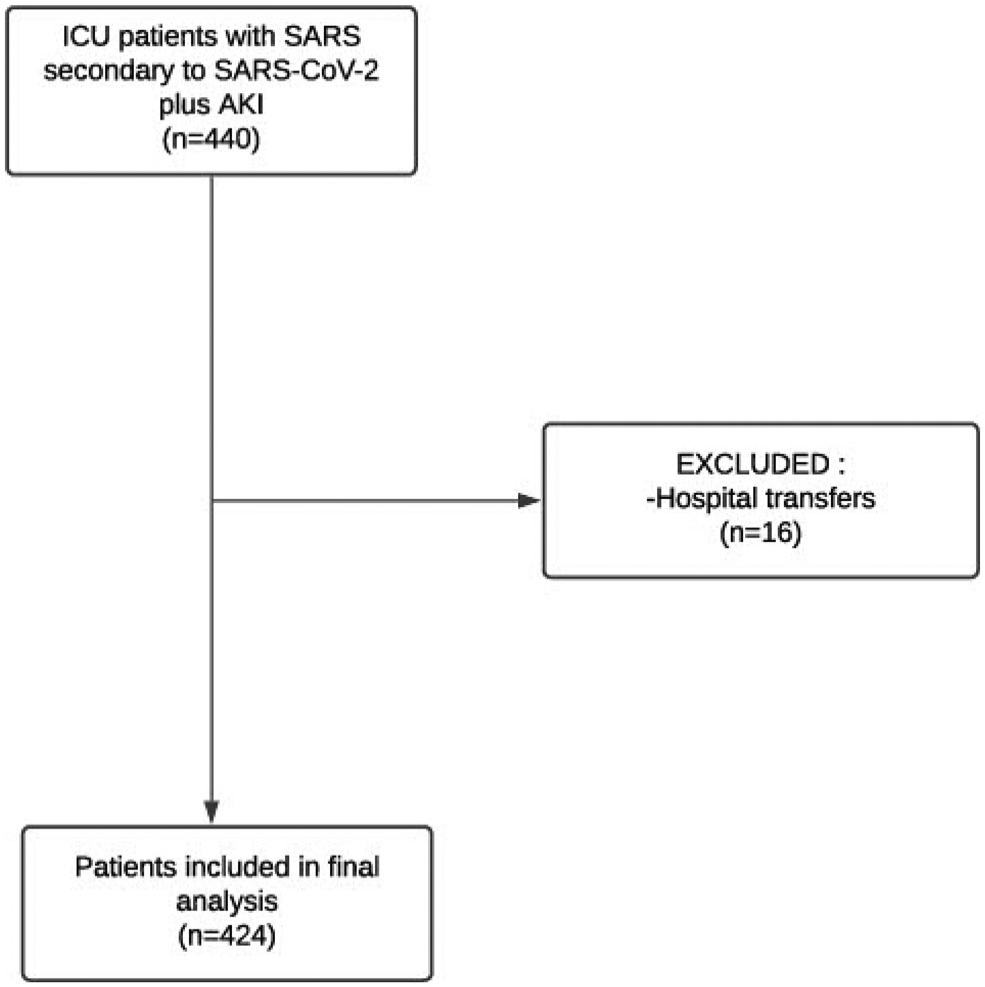
Flowchart of the study. SARS: severe acute respiratory syndrome.

The flow diagram of the study is shown in Figure 1. Between 8 March and 18 August 2020, the nephrology team was called upon to evaluate 739 patients with SARS and kidney failure. Of these, 485 patients tested positive for SARS-CoV-2, 440 of them classified as having AKI and 45 as having CKD. There were 88 patients who did not undergo RT-PCR for SARS-CoV-2 due to medical decisions or lack of tests. Sixteen patients who were transferred to other hospitals during the follow-up were excluded. The general characteristics of the 424 patients included in the study are shown in Table 1. The mean age was 66.42 ± 13.79 years. There was a male predominance (59.2%). It was possible to establish a severity profile using a quick SOFA score in 76% of our sample. The distribution of qSOFA by stage was 11.8% for qSOFA 1, 17.9% for qSOFA 2, and 70.3% for qSOFA 3. Regarding comorbidities, systemic arterial hypertension was present in 58.5%, diabetes mellitus in 39.9%, heart failure in 12.3%, obesity in 12.5%, and COPD in 13.7%. Mechanical ventilation was necessary for 90.3% of the patients, and 76.7% used vasopressors.

**Table 1.** General data of the sample (*n* = 424).

Variables	Results (%)
Age (years)	66.42 ± 13.79
Sex	
Male	251 (59.2%)
Female	173 (40.8%)
AKI etiology	
Sepsis	335 (84.2%)
Hypovolemia	45 (11.3%)
Obstructive	17 (4.3%)
Others	1 (0.3%)
Hemodialysis	335 (79%)
Time on dialysis (days)	3 [1–6]
Hemodialysis modality	
Prolonged	277 (82.4%)
Short	59 (17.6%)
Mechanical ventilation	383 (90.3%)
PEEP (cmH_2_O)	10.40 ± 2.45
Vasopressors	325 (76.7%)
qSOFA	
1	38 (11.8%)
2	58 (17.9%)
3	227 (70.3%)
Comorbidities	
Obesity	53 (12.5%)
Hypertension	248 (58.5%)
Diabetes mellitus	169 (39.9%)
COPD	58 (13.7%)
Heart failure	52 (12.3%)
Cancer	38 (9%)
KDIGO	
1	20 (4.7%)
2	79 (18.6%)
3	325 (76.6%)
Death	382 (90.1%)

Data are expressed as mean ± standard deviation or median (interquartile range).

COPD: chronic obstructive pulmonary disease; vasopressor (norepinephrine, epinephrine, dopamine and dobutamine); PEEP: positive end-expiratory pressure; qSOFA: quick Sepsis Related Organ Failure Assessment.

### In-hospital mortality and associated variables

Overall in-hospital mortality was 90.1%, without a significant difference among the hospitals. Univariate analysis for death (Table 2) showed that the mean age of nonsurvivors was significantly higher (66.9 ± 13.60 versus 62.07 ± 14.82 in survivors; *p* = 0.03). However, age was not a significant predictor of mortality in logistic regression, perhaps because both survivors and non-survivors had a statistically different but not widely separated age distribution. AKI secondary to sepsis was higher in nonsurvivors (89.7% versus 35%; *p* < 0.001). Patients on mechanical ventilation were disproportionately nonsurvivors (95% versus 47.6%; *p* < 0.001), as were those who needed vasopressors (81.2% versus 35.7%; *p* < 0.001), those who needed dialysis (82.7% versus 45.2%; *p* < 0.001), and those in KDIGO stage 3 (79.3% versus 52.4%; *p* = 0.002). In the logistic regression analysis (Table 3), the need for mechanical ventilation (OR = 8.44; CI 3.43–20.74; *p* < 0.001) and the need of vasopressors (OR = 2.93; CI 1.28–6.71; *p* = 0.01) were significantly associated with mortality. Other variables tested, such as KDIGO stage 3 (OR = 1.43; CI 0.59–3.45; *p* = 0.42) and age (OR = 0.99; CI 0.96–1.02; *p* = 0.09), were not independently associated with mortality.

**Table 2.** Distribution of variables according to mortality.

	Nonsurvivors (*n* = 382)	Survivors (*n* = 42)	*p*
Age (years)	66.90 ± 13.60	62.07 ± 14.82	0.03
Sex			0.96
Male	226 (59.2%)	25 (59.5%)	
Female	156 (40.8%)	17 (40.5%)	
AKI etiology			<0.001
Sepsis	321 (89.7%)	14 (35%)	
Hypovolemia	26 (7.3%)	19 (47.5%)	
Obstructive	11 (3.1%)	6 (15%)	
Others		1 (2.5%)	
Hemodialysis	316 (82.7%)	19 (45.2%)	<0.001
Time on dialysis (days)	2 [1–6]	23 [6–47]	<0.001
Hemodialysis modality			<0.001
Prolonged	269 (84.9%)	8 (42.1%)	
Short	48 (15.1%)	11 (57.9%)	
Mechanical ventilation	363 (95%)	20 (47.6%)	<0.001
PEEP (cmH_2_O)	10.45 ± 2.46	9.39 ± 2.03	0.07
Vasopressors	310 (81.2%)	15 (35.7%)	<0.001
qSOFA			<0.001
1	22 (7.6%)	16 (45.7%)	
2	47 (16.3%)	11 (31.4%)	
3	219 (76%)	8 (22.9%)	
Comorbidities			
Obesity	50 (13.1)	3 (7.1%)	0.19
Hypertension	220 (57.6%)	28 (66.7%)	0.16
Diabetes mellitus	152 (39.8%)	17 (40.5%)	0.52
COPD	52 (13.6%)	6 (14.3%)	0.52
Heart failure	44 (11.5%)	8 (19%)	0.12
Cancer	36 (9.4%)	2 (4.8%)	0.24
K^+^ plasmatic (mEq/L)	4.95 ± 1.12	4.96 ± 0.88	0.96
KDIGO			0.002
1	16 (4.2%)	4 (9.5%)	
2	63 (16.5%)	16 (38.1%)	
3	303 (79.3%)	22 (52.4%)	

Data are expressed as mean ± standard deviation or median (interquartile range).

COPD: chronic obstructive pulmonary disease; vasopressor (norepinephrine, epinephrine, dopamine and dobutamine); PEEP: positive end-expiratory pressure; qSOFA: quick Sepsis Related Organ Failure Assessment.

**Table 3.** Independent risk factors for mortality and KDIGO stage 3 (logistic regression).

	Mortality		KDIGO 3	
	OR	95% CI	OR	95% CI
Mechanical ventilation	8.44	3.43–20.74	2.60	1.23–5.45
Vasopressors	2.93	1.28–6.71	1.95	1.12–3.99
KDIGO 3	1.43	0.59–3.45		
Dialysis	2.16	0.88–5.32		
Age (years)	0.99	0.96–1.02		

OR: odds ratio; CI: confidence interval.

### Renal outcomes

The distribution of AKI by stage was 4.7% for KDIGO stage 1, 18.6% for KDIGO stage 2, and 76.6% for KDIGO stage 3. Most of the patients (93%) developed AKI in the first week of hospitalization. It was possible to establish AKI etiology in 398 patients (93.8%). Regarding AKI etiologies, the vast majority (84.2%) were sepsis-associated. AKI with dialysis indication (AKI-RRT) occurred in 79% of the patients, and the prolonged modality was the most frequent (82.4%). The frequency of AKI-RRT was 40% in KDIGO 1, 49.4% in KDIGO 2, and 88.6% in KDIGO stage 3 (*p* < 0.001). Regarding time on dialysis, 39% of the patients died within 24 h of undergoing the first dialysis session. Renal recovery of patients who were discharged was considered complete in 19 (46.34%), partial in 19 (46.34%) and progression to KFRT in 3 (7.31%).

### Severe AKI (KDIGO 3) and associated variables

Univariate analysis for the severity of AKI (Table 4) showed that the need for mechanical ventilation was more frequent in KDIGO stage 3 versus KDIGO stage 1 (93.5% versus 80%; *p* < 0.001), as were need of vasopressors (80.9% versus 70%; *p* < 0.001), sepsis-associated AKI (91.1% versus 47.4%; *p* < 0.001) and death (93.2% versus 80%; *p* < 0.001). The frequency of mechanical ventilation among those with AKI-RRT was higher than those who did not require dialysis (95.5% vs. 70.8%, *p* < 0.001). Independent risk factors for severe AKI (KDIGO stage 3) were the need for mechanical ventilation (OR = 2.60; CI 1.23–5.45; *p* = 0.01) and the need for vasopressors (OR = 1.95; CI 1.12–3.99; *p* = 0.01).

**Table 4.** Distribution of variables according to KDIGO.

	KDIGO 1 (*n* = 20)	KDIGO 2 (*n* = 79)	KDIGO 3 (*n* = 325)	*p*
Age (years)	69.68 ± 14.14	65.17 ± 13.81	66.76 ± 13.59	0.38
AKI etiology				<0.001
Sepsis	9 (47.4%)	48 (64%)	278 (91.1%)	
Hypovolemia	10 (52.6%)	19 (26.6%)	16 (5.2%)	
Obstructive		6 (8%)	11 (3.6%)	
Others		1 (1.3%)		
Hemodialysis	8 (40%)	39 (49.4%)	288 (88.6%)	<0.001
Time on dialysis (days)	2 [1–11]	4 [1–9]	2 [1–6]	0.89
Hemodialysis modality				0.19
Prolonged	8 (100%)	30 (75%)	239 (83%)	
Short		10 (25%)	49 (17%)	
Mechanical ventilation	16 (80%)	63 (79.7%)	304 (93.5%)	<0.001
PEEP (cmH_2_O)	9.87 ± 1.92	10.40 ± 2.45	10.44 ± 2.48	0.68
Vasopressor	14 (70%)	48 (60.8%)	263 (80.9%)	<0.001
qSOFA				0.07
1	3 (17.6%)	11 (19.6%)	23 (9.2%)	
2	4 (23.5%)	13 (23.2%)	41 (16.5%)	
3	10 (58.8%)	32 (57.1%)	185 (74.3%)	
Comorbidities				
Obesity	2 (10.5%)	13 (17.8%)	38 (11.7%)	0.48
Hypertension	13 (65%)	54 (68.4%)	181 (55.7%)	0.10
Diabetes mellitus	12 (60%)	38 (48.1%)	119 (36.6%)	0.02
COPD	4 (20%)	11 (13.9%)	43 (13.2%)	0.69
Heart failure	4 (20%)	17 (21.5%)	31 (9.5%)	0.08
Cancer	3 (15%)	10 (12.7%)	25 (7.7%)	0.23
K^+^ plasmatic (mEq/L)	4.41 ± 1.10	4.79 ± 0.88	5.03 ± 1.13	0.02
Death	16 (80%)	63 (79.7%)	303 (93.2%)	<0.0001

Data are expressed as absolute number and percentage, mean ± standard deviation or median (interquartile range).

COPD: chronic obstructive pulmonary disease; vasopressor (norepinephrine, epinephrine, dopamine and dobutamine); PEEP: positive end-expiratory pressure; qSOFA: quick Sepsis Related Organ Failure Assessment.

## Discussion

In this study, involving only critical patients with SARS and AKI due to SARS-CoV-2 infection, most of the patients were on mechanical ventilation (90.3%), and had high in-hospital mortality (90.1%). Compared to other similar studies, widely varied mortalities were reported (23.8–97.2%), probably resulting from different characteristics of the samples [3,4]. It is noteworthy to mention that data about mortality in the subgroup of patients who developed AKI and required mechanical ventilation were not available in most previous studies. Another important aspect to be considered in the context of mortality is the significant difference in the frequency of the need for critical care, which varies from 23.8% to 100% [2–5,12–14]. Richardson et al. assessed the outcomes of 2634 in-hospital patients with COVID-19. Only 373 of their patients (14.2%) were treated in the ICU, 320 patients (12.2%) required mechanical ventilation, and 520 patients (22.2%) developed AKI. The overall mortality was 21%. However, the in-hospital AKI-RRT mortality rate in their study was 83.8%, and the mortality of those who received mechanical ventilation was 88.1% (*n* = 282), suggesting the significance of these two variables in determining mortality [4]. Yang et al. analyzed 52 critically ill adult patients with COVID-19 and observed a prevalence of AKI, AKI-RRT, and MV of 29%, 17%, and 42%, respectively. The authors found overall 28-day mortality of 61.5% and AKI-RRT mortality of 88% [12]. Mohamed et al. published data from 575 patients with COVID-19, 30% of whom were in the ICU. The authors observed an AKI prevalence of 28% and 63% of these patients required mechanical ventilation. The in-hospital mortality rate for those with AKI-RRT was 72%. In line with our findings, they found that the frequency of mechanical ventilation among those with AKI-RRT was higher compared to those who did not require RRT (82% vs. 39%; *p* < 0.001) [14]. It must be stressed that even in the less severe forms of AKI described in our study (twenty KDIGO stage 1 patients), we observed a high frequency of dialysis requirement (40%) and a high rate of mortality (80%). One plausible reason for these findings is that many of our patients in KDIGO 1 and 2 were already oliguric, with low PaO2/FiO2 ratio and with a very positive fluid balance or even with disproportionately high potassium levels, a phenomenon that has been described in COVID-19-associated AKI [15].

Conversely, patients with other infections with the potential to induce AKI, such as leptospirosis, presented a lower frequency of AKI-RRT, as well as lower mortality, when in KDIGO stage 1. One possible explanation could be the higher frequency of reversible factors in leptospirosis-associated AKI, such as hypovolemia [16].

Another plausible explanation for such high mortality in COVID-19-associated AKI, even in less severe forms (KDIGO 1), could be its etiology and pathophysiology. There are some potential mechanisms underlying these differences, such as endothelial dysfunction, cytokine storm (sepsis), rhabdomyolysis, coagulopathy, drug-induced nephrotoxicity, and even direct tubular lesion by the virus, but all of these factors could be present in the same patient [17,18]. In our study, the most frequent etiology (84.2%) was sepsis-associated AKI. Expressive mortality in sepsis-associated AKI has been described in several cohort studies. For example, the Sepsis Occurring in Acutely Ill Patients (SOAP study) included 3147 individuals from 198 European ICUs. They described a 37% prevalence of sepsis and 51% AKI in those patients, with an associated ICU mortality of 41% [19]. It has been suggested that patients with COVID-19 have a higher incidence of AKI, more severe forms of AKI, and more need for ICU beds, mechanical ventilation, and RRT than patients with other causes of AKI. Additionally, patients with AKI who are positive for COVID-19 are less likely to recover kidney function and more likely to experience in-hospital death than patients with AKI of other etiologies at the same AKI stage [5].

Data about AKI in COVID-19 patients in Brazil are scarce. In a recent study that included 101 patients admitted to the ICU of a private hospital, AKI was diagnosed in 50.2%, out of which 38.6% were in KDIGO stage 3, and AKI-RRT was diagnosed in 33.6%. 69.3% and 65.3% of AKI patients required mechanical ventilation and vasopressors, respectively. In addition, the 60-day AKI mortality and 60-day AKI-RRT mortality rates were 23.8% and 35.3%, respectively [3]. Possible reasons for this much lower mortality than has been found in other studies could be the lower prevalence of KDIGO stage 3, the short time interval between COVID-19 symptom onset and ICU admission (only 1 day), and the study setting (private medical service with better resources). Recently, Ranzani et al. published the largest cohort of patients with COVID-19 from the Brazilian Nationwide Surveillance Database, the first 250,000 patients hospitalized with COVID-19 in public and private services from all regions of the country, and demonstrated another reality, thus, the ICU mortality being 59%, and the in-hospital mortality of those who required mechanical ventilation at 80%. However, the data regarding AKI and RRT was not available [20]. It has to be noted, therefore, that our sample has different characteristics from most previous studies. For instance, all the patients in our study were under intensive care due to SARS and had a diagnosis of AKI. Furthermore, 76.6% presented the most severe form of AKI, 84.2% sepsis-associated AKI, 90.3% required mechanical ventilation, 76.7% required vasopressors, and 79% underwent dialysis. Additionally, at the beginning of the pandemic, the world faced a shortage crisis with a lack of hospital beds and supplies, especially in intensive care units, and uncertainties about how to deal with a new disease and the real benefits of some drugs. This scenario was not different in Brazil, and this cohort covers exactly this period. Recently, Gupta et al. described that admission to a hospital with fewer ICU beds and in regions with a greater regional density of COVID-19 were predictors of mortality in American hospitals [21]. Although the regional density of COVID-19 was not addressed in the period of our study, the majority of primary care units in our city were characterized by overcrowding, and long waiting times (patients probably waited from hours to a few days to get an ICU bed). We believe that the extremely high mortality we observed in our patients may have been the result of local factors such as the limited resources of public hospitals in the middle of the pandemic and the regional density of COVID-19 as described by other authors. In the present study, 90.3% of the patients were on mechanical ventilation, and this variable was the most important independent risk factor for mortality and severe AKI (KDIGO 3). In a similar study by Hirsch et al., it was observed that 96.8% of patients with COVID-19 requiring RRT were on mechanical ventilation [13]. The aforementioned evidence reinforces the hypothesis of ‘lung–kidney cross-talk’ in critically ill patients, as demonstrated in several studies [22,23]. Johanes et al. reported that mechanical ventilation was associated with a threefold increased risk of AKI in critically ill patients [24]. In another study, Walcher et al. compared patients with AKI and CKD hospitalized in intensive care requiring dialysis therapy and demonstrated after a multivariate analysis that the need for mechanical ventilation was the single driving factor associated with increased mortality (OR = 3.4) [25]. In a subgroup analysis, Richardson et al. demonstrated that mortality in patients with COVID-19 who received mechanical ventilation could reach 97.2% in patients older than 65 years [4].

In the present study despite severe AKI and the dialysis need were more frequent in non-survivors, they were not independent risk factors for death. This intriguing finding deserves further scrutiny since AKI severity is a well-established risk factor for mortality in critical patients. We believe that our patients were in such a critical condition that AKI severity and need for dialysis were supplanted by the other COVID-19 complications (severe hemodynamic instability, SARS and severe thrombotic events). Reinforcing this hypothesis, many of the patients who were part of our cohort underwent only one or two dialysis sessions before dying, a fact that can be confirmed by the shorter time on dialysis in the non-surviving group. Additionally, our sample has a high percentage of patients with hypertension (58.5%) and diabetes (39.9%), which are well-known risk factors for more serious manifestations of the disease [4].

The present study had several strengths. First, to the best of our knowledge, this is the largest cohort of critically ill patients with AKI during the COVID-19 pandemic studied in Brazil, which is the third-highest country in terms of the number of confirmed cases [26]. In addition, it describes the reality of public hospitals in our country, and hence the findings described here can help in the future management of similar patients as well as planning for ICU care. There were some limitations with our study: (1) it was an observational analysis; (2) we did not have access to adequate urinalysis results to describe the main findings on the urine contents; (3) we used the lowest inpatient serum creatinine as a reference for AKI.

In conclusion, this multicenter cohort study has demonstrated that critical patients with SARS and AKI due to COVID-19 had high mortality. Mortality was largely determined by the need for mechanical ventilation and need of vasopressors rather than by AKI severity in these severely ill patients.
